# Die transossäre Rekonstruktion der Trizepssehnenruptur: Operationstechnik

**DOI:** 10.1007/s00113-021-01007-5

**Published:** 2021-05-22

**Authors:** Nael Hawi, Sam Razaeian, Christian Krettek

**Affiliations:** Unfallchirurgische Klinik, Medizinische Hochschule Hannover, Carl-Neuberg-Str. 1, 30625 Hannover, Deutschland

**Keywords:** Ellenbogen, Trizepssehne, Rekonstruktion, Transossär, Nahttechnik, Elbow, Triceps tendon, Reconstruction, Transosseous, Suturing

## Abstract

**Operationsziel:**

Transossäre Rekonstruktion der Trizepssehne.

**Indikation:**

Sämtliche Trizepssehnenrupturen, die eine spannungsfreie Rekonstruktion erlauben.

**Kontraindikation:**

Retrahierte Trizepssehnenrupturen, die nach Mobilisation keine spannungsfreie Rekonstruktion erlauben.

**Operationstechnik:**

Durch 2 sich kreuzende transossäre Kanäle erfolgt das Durchfädeln eines nichtresorbierbaren Fadens. Direkt im Footprint erfolgt zudem das Setzen eines Fadenankers. Durch den primären Faden erfolgt nach transossärem Shutteln am Footprint beginnend das Durchflechten der Sehne in Krackow-Nahttechnik und, erneut am Footprint angekommen, das erneute transossäre Shutteln sowie das körperferne Verknoten. Mit dem ersten Fadenpaar des Ankers erfolgen in ähnlicher Weise das Armieren der Sehne und anschließend das intratendinöse Verknoten. Durch ein Verknoten des zweiten Fadenpaares des Ankers auf den primären körperfernen Knoten kann der Anpressdruck auf das Avulsionsfragment erhöht werden. Alternativ kann mit den Fäden des Fadenankers eine Mason-Allen Naht-durchgeführt werden.

**Weiterbehandlung:**

Die ersten 6 Wochen erfolgt die Nachbehandlung in einer „ROM brace“ mit einem stufenweisen Freigeben der Flexion. Nach 6 Wochen freie Flexion. Beginn mit Kräftigungsübungen nach 12 Wochen.

**Ergebnisse:**

Autoren beschreiben gute Ergebnisse nach operativer Versorgung von Trizepssehnenrupturen. Im vorliegenden Fall wird 6 Monate postoperativ, ungeachtet von einem in der Literatur beschriebenen möglichen Extensionsdefizit, ein exzellentes Outcome mit freiem Bewegungsausmaß erreicht.

## Abstract (en)

**Objective:**

Transosseous reconstruction of the triceps tendon.

**Indications:**

All tears of the triceps tendon that allow a tension-free reconstruction.

**Contraindications:**

Retracted triceps tendon tears that do not allow a tension-free reconstruction after mobilization.

**Surgical technique:**

Two nonabsorbable sutures are threaded through two crossing transosseous canals. In addition, an anchor is placed directly in the footprint. Using the first nonabsorbable suture the tendon is sutured using the Krackow technique and subsequently shuttled transosseously distally and knotted there with its other part. Using the first suture pair of the anchor the tendon is sutured in a similar manner and knotted within the tendon. With the second pair pressure to the avulsion fragment can be increased by knotting it distally to the first knot. Alternatively, sutures of the suture anchor can be used with a Mason-Allen technique.

**Postoperative management:**

For the first 6 weeks a ROM brace is applied with gradual release of flexion. Free flexion after 6 weeks is allowed. Beginning of strengthening exercises after 12 weeks.

**Results:**

Authors have reported good results after surgical treatment of triceps tendon ruptures. Regardless of possible extension deficits described in the literature, in the case presented an excellent postoperative outcome with a free range of motion was achieved.

## Vorbemerkung

Die Trizepssehnenruptur ist eine seltene Verletzung und stellt weniger als 1 % aller Sehnenverletzungen dar [1–3].

**Figure 1:**
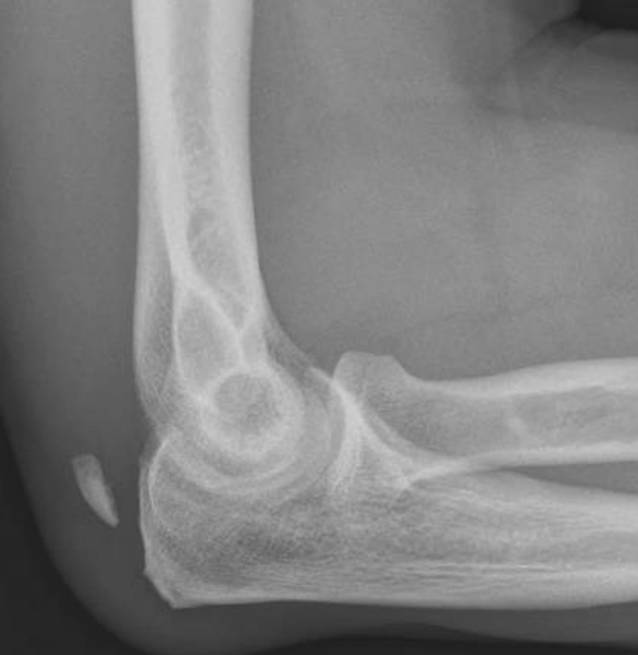

Die Schwere der Trizepssehnenverletzung kann variieren und reicht von der Partial- bis hin zur kompletten Ruptur bzw. v. a. zu Avulsionsverletzungen ([4]; Abb. 1).

Seltener liegt die Läsion im intramuskulären oder myotendinösen Bereich [5–7]. Üblicherweise führt der Sturz auf den ausgestreckten Arm zu dieser Verletzung, wobei auch das direkte Trauma auf die obere Extremität als Ursache beschrieben wurde [8–10]. Zudem beschreibt die Literatur ein vermehrtes Auftreten bei Gewichthebern und bei Konsumenten von anabolen Steroiden [11–14].

Nicht selten führt das Unterschätzen des Verletzungsausmaßes bzw. die erschwerte und verzögerte Diagnosestellung zu einer Verzögerung bei der chirurgischen Behandlung [14]. Eine gründliche körperliche Untersuchung ist erforderlich, um ein Nichterkennen dieser Verletzung zu vermeiden. Durch die konventionell radiologische Bildgebung lassen sich Avulsionsverletzungen erkennen ([15]; Abb. 1). Sowohl die sonographische Untersuchung als auch die MRT-Bildgebung können die Diagnose bestätigen. Eine frühe Diagnosestellung erhöht die Wahrscheinlichkeit der direkten Rekonstruktionsmöglichkeit [16].

Patienten berichten oft über ein schmerzhaftes Knallen am Ellenbogen. In der körperlichen Untersuchung zeigen sich ein lokaler Schmerz, Schwellung und Hämatom. Auch kann die Palpation eines Defekts an der Trizepssehne möglich sein. Klinisch kann sich die Extension gegen Widerstand im Ellenbogengelenk schmerzhaft und mit einem Kraftverlust zeigen. Vor allem das Strecken des Ellenbogens gegen Widerstand bei um 180 Grad elevierter Schulter zeigt sich auffällig. Als Provokationstest ist der modifizierte Thompson-Test beschrieben. Ähnlich zur Achillessehnenruptur führt die Kompression des Trizepsbauches zu einer Extension im Ellenbogengelenk. Vorzugsweise befindet sich der Patient hierfür in Bauchlage mit hängendem Unterarm [15, 17].

Prinzipiell orientiert sich die Therapie an dem Ausmaß der Ruptur, der Lokalisation, dem funktionellem Defizit sowie dem Aktivitätsausmaß des Patienten. Partialrupturen werden meist der konservativen Therapie zugeführt; komplette Rupturen oder Avulsionsverletzungen werden üblicherweise chirurgisch refixiert [4, 18]. Die meisten beschriebenen Techniken verwenden Fadenanker, transossäre Bohrkanäle bzw. Tunnel und/oder primäre Nahttechniken, wobei weiterhin kein Konsens über die ideale Versorgungsstrategie besteht [14, 18–20].

## Operationsprinzip und -ziel

Transossäre bzw. transossär-äquivalente Reinsertion der Trizepssehne.

## Vorteile

Sichere und flächige Reinsertion der Sehne an ihrer ursprünglichen Insertionsstelle.

Zugrichtung der Fäden in Richtung der Zugrichtung der Sehne.

Durch die Bohrkanäle Zufluss von Stammzellen an die Rekonstruktionsunterfläche.

Vermeiden einer intraartikulären Perforation durch die distale Ankerbesetzung im Rahmen einer üblichen zweireihigen Versorgung.

Erhöhung des Anpressdruckes der Sehne auf den Footprint durch das Verwenden eines Fadenankers in selbigem.

## Nachteile

Offene Technik mit dementsprechend bedingter Zugangsmorbidität.

## Indikationen

- Komplettrupturen,
- Partialrupturen mit Extensionsdefizit,
- Versagen der konservativen Therapie,
- keine Altersbeschränkung, Indikation abhängig vom Retraktionsgrad, der Sehnenqualität und der entsprechenden Klinik und Motivation des Patienten.

## Kontraindikationen

Keine ausreichende Mobilisierung der Trizepssehne möglich zur spannungsarmen Reinsertion.

Die Versorgung von chronischen Rupturen, retrahierten Rupturen und/oder muskulären Atrophien erfordert ggf. das Verwenden von Augmentationstechniken.

Allgemein bedingte (beispielsweise kardiale) und/oder lokale Gründe (Infektion).

## Patientenaufklärung

Erläuterung der vorliegenden Pathologie, idealerweise am Modell.

Keine spontane Sehnenheilung möglich.

Allgemeine chirurgische Risiken:

- Hämatom,
- Wundheilungsstörung,
- Wundinfekt,
- Nervenverletzung bei Präparation (N. ulnaris, N. radialis),
- Bewegungseinschränkung,
- komplexes regionales Schmerzsyndrom (CRPS),
- Thrombose.

Spezifische Folgen:

- Bewegungseinschränkung mit v. a. verbleibendem Streckdefizit,
- Kraftminderung,
- materialassoziierte Beschwerden (Knoten unter der Haut palpabel), materialassoziierte Komplikationen (Lockerung und Ausbrechen der verwendeten Anker, Durchschneiden der Fäden v. a. bei degenerativen Veränderungen der Sehne),
- prolongierte Ausheilungsdauer,
- Arbeitsunfähigkeit je nach beruflicher Tätigkeit zwischen 3 und 6 Monaten,
- Reruptur,
- Nahtdehiszenz,
- Nahtinsuffizienz,
- muskulärer Schmerz im Bereich des Trizepsmuskels,
- Restbeschwerden.

## Operationsvorbereitungen

- Anamnese mit Genese der Verletzung (akut vs. chronisch, Dauer, Schmerz),
- klinische Untersuchung mit palpabler Delle, Kraftverlust bei Extension gegen Widerstand, Bewegungsausmaß,
- Röntgen mit Ellenbogen (a.-p. und seitliche Darstellung, *cave*: knöcherne Avulsionsverletzung (knöcherner Ausriss des Sehnenansatzes, „flake of bone“)),
- Sonographie zum Nachweis einer Ruptur,
- MRT zur Beurteilung der Retraktion und der Muskelqualität.

## Instrumentarium

- Bohrmaschine (2,5-mm-Bohrer),
- Fadenfasshaken,
- oszillierende Säge oder Kugelfräse zum Anfrischen des Footprint,
- nichtresorbierbare Nahtfäden (z. B. FiberWire, Fa. Arthrex, Naples, Florida, USA),
- Fadenanker (z. B. JuggerKnot Soft Anchor, Fa. Zimmer, Warsaw, Indiana, USA, oder Y‑Knot RC, Fa. Conmed, Utica, New York, USA).

## Anästhesie und Lagerung

**Figure 2:**
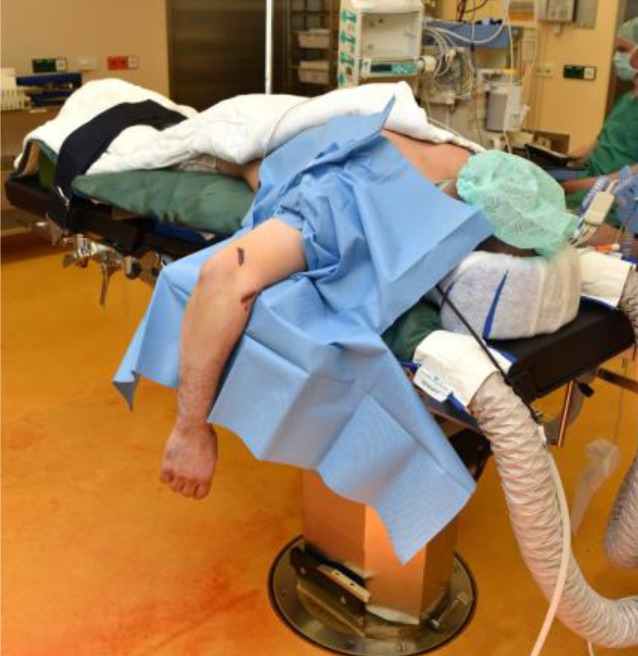

- Intubationsnarkose, wahlweise mit einem interskalenären Schmerzkatheter,
- Lagerung wahlweise in Seiten- oder Bauchlagerung (Abb. 2) mit ausgelagertem Arm auf einem kurzen Armtisch,
- Anlage einer Blutsperre kann hilfreich sein, aber u. U. die Mobilisation bzw. Reposition der Trizepssehne erschweren.

## Operationstechnik

### Schritt 1 (Abb. 3)

Bogenförmiger Hautschnitt, beginnend radial der Olekranonspitze knapp proximal des Olekranons, reichend nach distal in Richtung der proximalen Ulna. Hierbei Vermeidung der Verletzung des N. ulnaris. Nach Durchtrennen der Subkutis wird die Trizepssehne dargestellt bzw. der rupturierte Bereich dargestellt.

**Figure 3:**
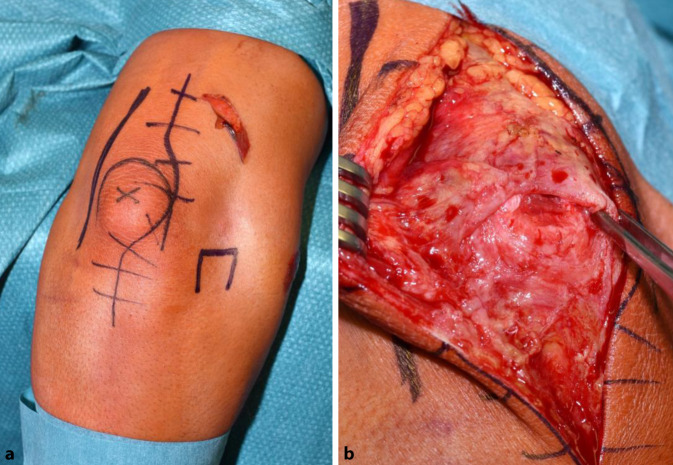

### Schritt 2 (Abb. 4)

Falls präparatorisch sinnvoll, erfolgt die Darstellung des N. ulnaris. Eine Verlagerung ist üblicherweise nicht erforderlich.

**Figure 4:**
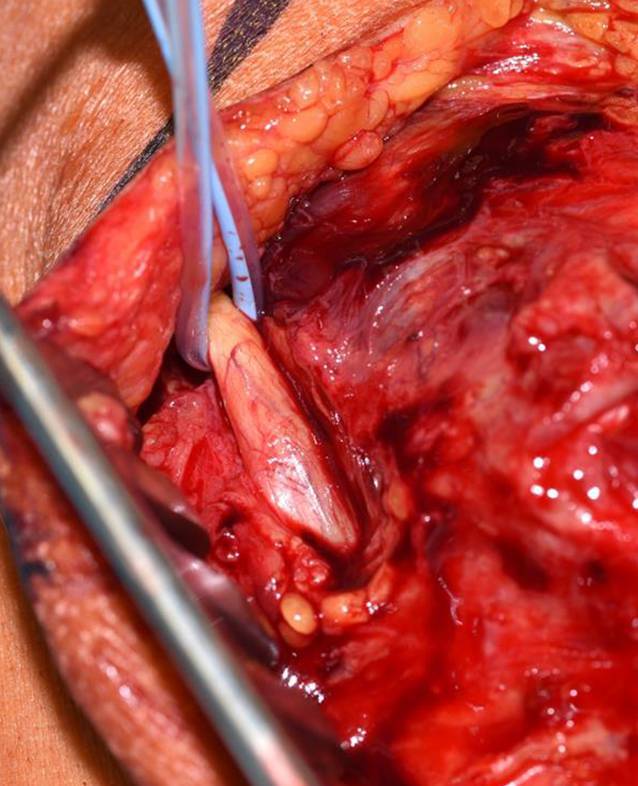

### Schritt 3 (Abb. 5)

Es erfolgt das Débridement des Trizepssehnenstumpfes und des Footprint. Dieses kann sowohl mit dem Lüer als auch mit der Kugelfräse und/oder der oszillierenden Säge erfolgen. Evaluierung des Mobilisierungsgrades der Sehne.

**Figure 5:**
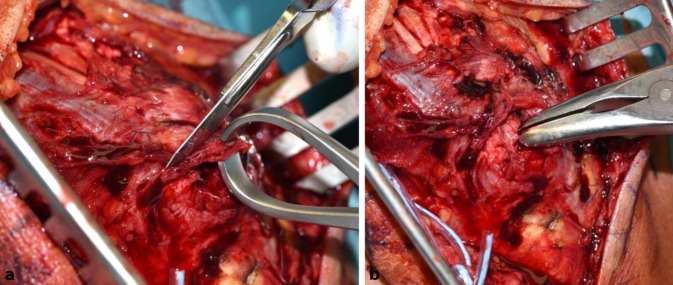

### Schritt 4 (Abb. 6)

Es werden nun 2 sich kreuzende Bohrkanäle (2,5 mm) gesetzt, beginnend mit dem ersten distal ulnar nach kranial radial und dem zweiten von kranial ulnar nach distal radial. Dadurch Vermeidung einer Verletzung des N. ulnaris. Setzen eines Ankers zentral im Olekranon im Bereich des Footprint. Es empfiehlt sich aufgrund der Knochendichtigkeit im Bereich des Olekranons, den Anker vorzubohren.

**Figure 6:**
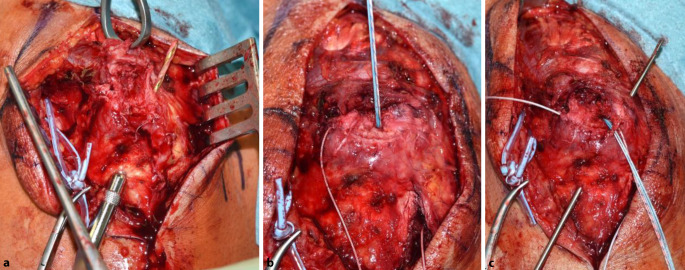

### Schritt 5 (Abb. 7)

Der Ellbogen wird nun gestreckt und die Sehne reponiert. Ein Faden wird mithilfe eines Fadenfasshakens durch einen Bohrkanal geführt. Mit dem Fadenende am Footprint wird die Sehne ulnar und radial beispielsweise mittels Krackow-Nahttechnik armiert und anschließend vom Footprint durch den zweiten Bohrkanal von kranial nach distal geshuttelt. Die beiden Fadenenden, welche nun distal die Bohrtunnel verlassen, werden nun verknotet.

**Figure 7:**
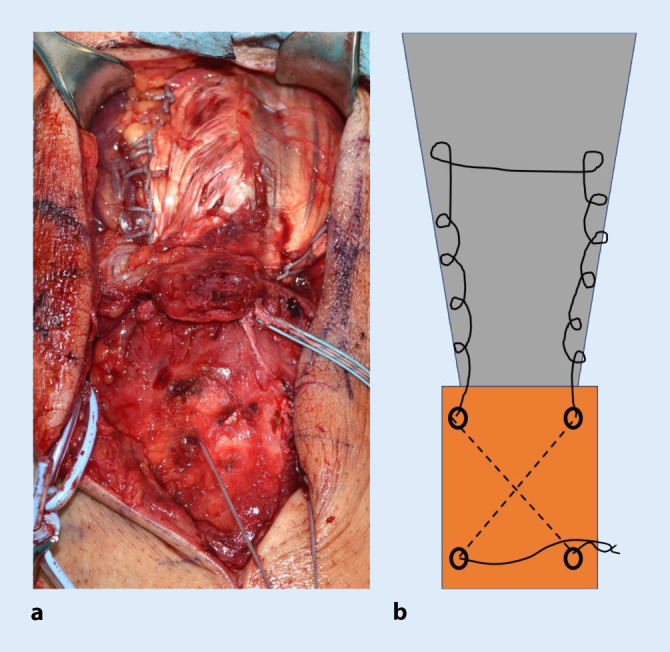

### Schritt 7

**Figure 8:**
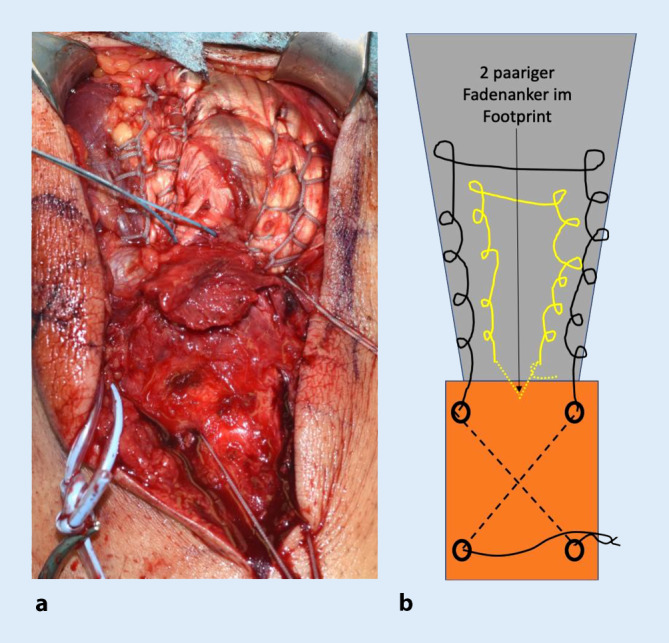

In ähnlicher Weise erfolgt das Vorgehen zentral mit dem ersten Fadenpaar des eingebrachten Ankers. Auch hier wird ulnarseits mittels Krackow-Nahttechnik die Sehne hochgenäht und radialseits erneut herunter zum Footprint. Sodann erfolgt das Verknoten am Footprint, wobei der Knoten in der Sehne versenkt wird. Der zentrale Anker bewirkt, flächigen Anpressdruck der Sehne auf den Footprint zu erzielen (Abb. 8).

**Figure 9:**
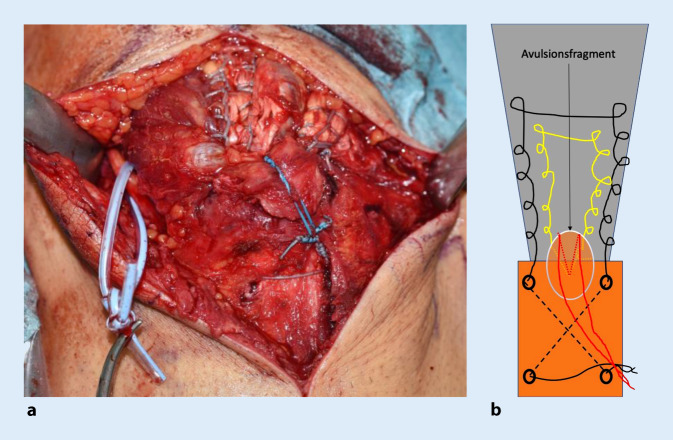

Bei der Verwendung eines Ankers mit 2 Fadenpaaren kann mit dem zweiten Fadenpaar das distal zu liegen kommende Knochenfragment nach distal gezügelt werden. Hierzu erfolgt das Verknoten auf den bereits distal gelegenen Knoten des primär eingebrachten freien Fadens (Abb. 9).

**Figure 10:**
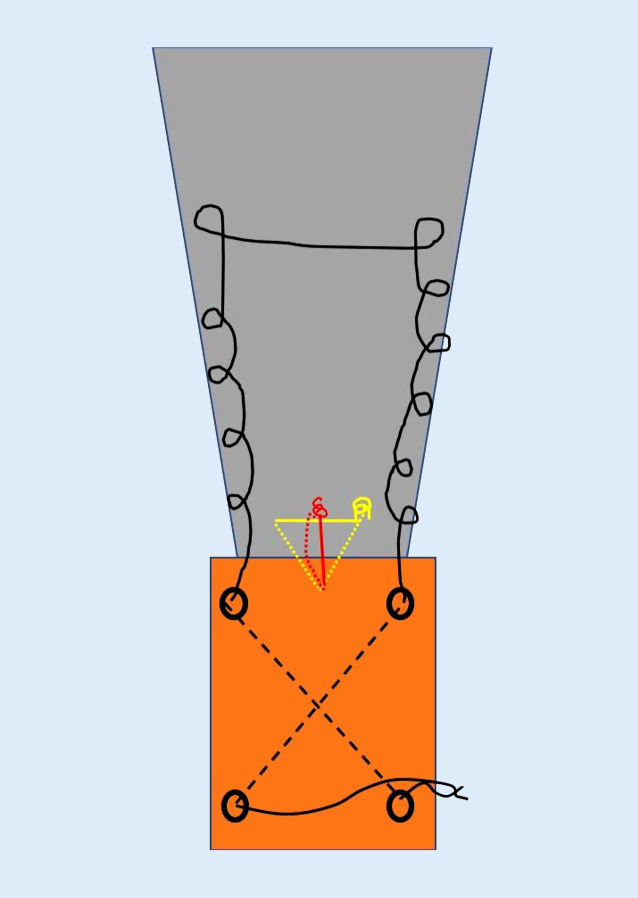

Alternativ kann eine Mason-Allen-Naht mit den 2 Fadenpaaren durchgeführt werden (Abb. 10).

### Schritt 8

Der Ellbogen wird nun gebeugt, um die Spannung der Sehne zu bewerten. Zeigt sich ein Klaffen der Sehne bei der Bewegung, sollte die Sehne durch weitere Fäden stabil armiert werden. Eine spannungsfreie Reinsertion der Sehne sollte sich nun zeigen.

## Postoperative Behandlung

**Figure 11:**
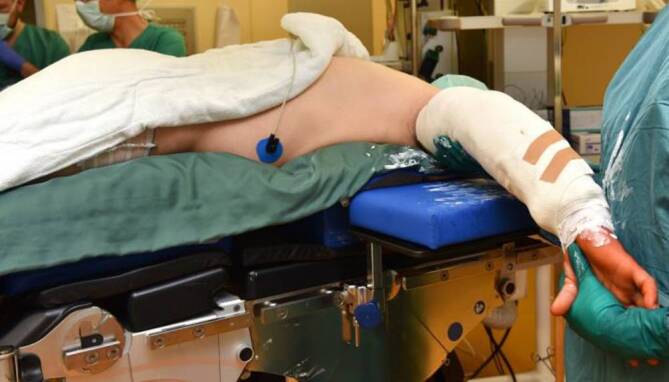

Im OP erfolgt das Anlegen einer Gipsschiene in Streckstellung (Abb. 11).

Am ersten postoperativen Tag erfolgt der Wechsel auf eine „ROM brace“ mit freier Streckung und limitierter Beugung auf 30 Grad für die ersten 2 Wochen, im Anschluss Erweiterung auf 60 Grad für die nächsten 2 Wochen und abschließend auf 90 Grad für weitere 2 Wochen. Nach 6 Wochen freie Flexion. Beginn mit Kräftigungsübungen nach 12 Wochen.

## Ergebnis

**Figure 12:**
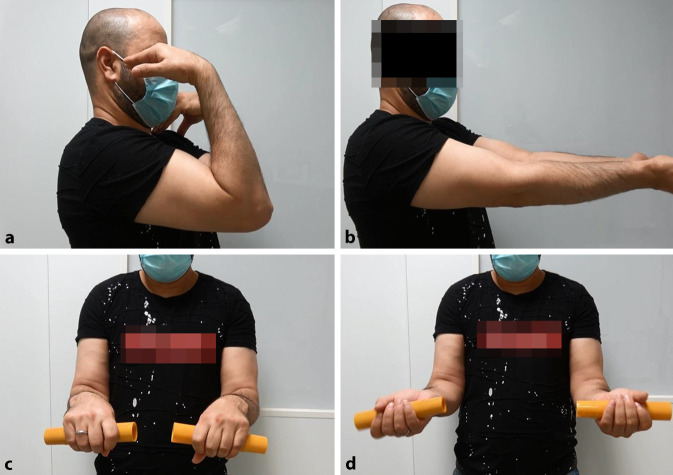

Sechs Monate postoperativ stellt sich der schmerzfreie 41-jährige Patient zur Verlaufskontrolle vor. Der Zugang ist reizlos verheilt. Eine Delle im Bereich der Trizepssehne ist nicht tastbar, und Konturunregelmäßigkeiten im Seitenvergleich sind nicht sichtbar. Der Patient berichtet zu diesem Zeitpunkt von einer subjektiven Ellenbogenfunktion von 100 % [21]. Der Mayo-Elbow-Performance-Score beträgt ebenfalls 100 %. Der Oxford-Elbow-Score beträgt 48 von 48 Punkten. Für die aktiven und passiven Bewegungsausmaße lassen sich seitengleiche, freie Bewegungsumfänge dokumentieren (Abb. 12).

In der isometrischen Kraftmessung der Extension (IsoForceControl EVO2 isometric dynamometer, Fa. Herkules Kunststoff Oberburg AG, Oberburg, Schweiz) am 90°-flektierten Ellenbogengelenk ergibt sich eine Maximalkraft von 100 N für die betroffene rechte Seite gegenüber 130 N für die linke Seite.

Der Patient ist vollintegriert in seinem Vollzeitjob als Lagerlogistiker bei einer großen Möbelhauskette und ist erneut auf seinem ursprünglichen Niveau beruflich tätig.

## Fehler, Gefahren, Komplikationen

Verletzung der Nn. ulnaris et radialis sowohl bei der Präparation als auch bei der Mobilisierung.

Wiederfinden der gekreuzten Bohrkanäle; hier kann das Vorschieben von Kirschner-Drähten mit dem stumpfen Ende hilfreich sein, um den transossären Kanal wiederzufinden.

Verbiegen oder Abbrechen des Fadenfasshakens im Bohrkanal.

Fadenbruch durch wiederholte transossäre Passage oder Manipulation durch den Fadenfasshaken.

Zu viel Nahtmaterial unterbindet die biologische Restdurchblutung, zu wenig reduziert die biomechanisch erforderliche Stabilität.

Vor allem bei subakuten oder chronischen Rupturen kann das Differenzieren zwischen Sehnengewebe und Narbe erschwert sein.

Kein zu aggressives Débridement der Kortikalis des Olekranons, da hierdurch die Stabilität des eingebrachten Ankers reduziert werden kann.

Es ist sehr wichtig, sich der Lokalisation der Nn. ulnaris et radialis bewusst zu sein und diese bei Bedarf darzustellen.

Falls intraoperativ eine direkte Reinsertion der Trizepssehne nicht mehr möglich ist, ist das Ausweichen auf ein Graft erforderlich.

## Diskussion

In der Literatur werden verschiedene operative Techniken für die Rekonstruktion der akuten, distalen Trizepssehnenruptur beschrieben. Neben Nahttechniken wie Bunnell- und Krackow-Naht zur Armierung der Sehne wird eine Reihe von möglichen Refixationstechniken genannt. Dazu gehören neben der transossären Refixation über 2 gekreuzte Bohrtunnel durch das Olekranon eine einfache Refixation über Fadenanker sowie eine knotenlose Doppelreihentechnik mit Verwendung von Fadenankern in Kombination mit sog. Faden-Tapes. Evidenzbasierte Therapieempfehlungen für die jeweiligen Techniken liegen nicht vor [18, 22].

Insgesamt zeigen publizierte Ergebnisse nach distaler Trizepssehnenrekonstruktion gute Ergebnisse. Zwar liefern die meisten retrospektiven Fallserien keine vergleichbaren quantitativen Daten bzw. Funktionsscores, lassen jedoch zuversichtliche Ergebnisse aus Kraft- und Beweglichkeitsmessungen ableiten [22]. So berichtet van Riet in seinem Patientenkollektiv mit 13 Patienten nach transossärer Refixation 7 Monate postoperativ von einer durchschnittlichen Extensionskraft von 57 % im Vergleich zur kontralateralen Seite sowie von einem Extensionsdefizit von 8 Grad mit einer durchschnittlichen Extensionskraft von 92 % im Vergleich zur kontralateralen Seite ein Jahr postoperativ [14]. Daten der nationalen Datenbank für Verletzungen bei American-Football-Spielern zeigten in den Jahren von 1991 bis 1996 11 akute Rupturen, die operativ versorgt wurden. Weder ein Bewegungsdefizit noch ein Kraftdefizit wurden in diesem Kollektiv beschrieben [1]. Auch Bava et al. beschrieben in ihrem Kollektiv mit 5 Patienten gute Ergebnisse nach Versorgung mittels Fadenankerrekonstruktion bei akuten Trizepssehnenrupturen [19].

Welche Refixationstechnik dabei das überlegenere Outcome liefert, wird in der Literatur kontrovers diskutiert. Die einzelnen Techniken sind unter biomechanischen Aspekten untersucht und gegeneinander verglichen worden. So konnten Carpenter et al. in ihrer Studie keine statistisch signifikanten Unterschiede in der Konstruktstabilität von transossären Refixationen gegenüber Fadenankerrefixationen in Doppelreihentechnik feststellen. Die Autoren hielten fest, dass vielmehr die Anzahl der Fäden bzw. Ankerreihen eine größere Rolle für die Stabilität als die reine Wahl der Technik zu spielen scheint. Des Weiteren suggerierten die Autoren mögliche signifikante Kostenunterschiede zuungunsten der Ankertechnik sowie eine größere Gefahr der intraartikulären Perforation [23, 24].

Horneff et al. konnten in ihrer retrospektiven Kohortenstudie keinen wesentlichen klinischen Unterschied in den beiden Techniken feststellen, wenn auch die Gruppe nach transossärer Refixation mit durchschnittlich 2,98 Punkten statistisch signifikant schlechter im DASH-Score abschnitt. Die Autoren schlussfolgerten, dass die Wahl der Refixationstechnik in Abhängigkeit von der jeweiligen Präferenzen des Chirurgen getroffen werden sollte [25].

Yeh et al. konnten hingegen in ihrer Kadaverstudie biomechanische Vorteile in der Doppelreihentechnik gegenüber einer transossären und einer einfachen Fadenankerrefixation sehen [26]. Mirzayan et al. beobachteten in ihrer retrospektiven Kohortenstudie sogar signifikant höhere Raten für Rerupturen (6,7 % vs. 0 %; *p* = 0,0244) und Folgeoperationen (9,5 % vs. 1,4 %; *p* = 0,026) sowie eine längere Hospitalisationsdauer (4,3 vs. 3,4 Monate; *p* = 0,0014) bei der Verwendung einer transossären Vorgehensweise [27].

Im vorliegenden Fall wurde eine Hybridtechnik angewendet, bei der eine transossäre Refixation mit einer Fadenankertechnik kombiniert wurde. 6 Monate postoperativ wird im vorliegenden Fall, ungeachtet von einem in der Literatur beschriebenen möglichen Extensionsdefizit, ein exzellentes Outcome mit freiem Bewegungsausmaß erreicht [28]. Autoren beschreiben ein Jahr nach der Versorgung eine Kraft von 80 % im Vergleich zur kontralateralen Seite und eine Festigkeit von 99 % [14, 16]. Trotz allem beschreibt die Literatur weiterhin eine hohe Rerupturrate mit bis zu 21 %.

## References

[1] Mair SD, Isbell WM, Gill TJ, Schlegel TF, Hawkins RJ (2004). Triceps tendon ruptures in professional football players. Am J Sports Med.

[2] Sierra RJ, Weiss NG, Shrader MW, Steinmann SP (2006). Acute triceps ruptures: case report and retrospective chart review. J Shoulder Elbow Surg.

[3] Anzel SH, Covey KW, Weiner AD, Lipscomb PR (1959). Disruption of muscles and tendons; an analysis of 1, 014 cases. Surgery.

[4] Strauch RJ (1999). Biceps and triceps injuries of the elbow. Orthop Clin North Am.

[5] Aso K, Torisu T (1984). Muscle belly tear of the triceps. Am J Sports Med.

[6] Bach BR, Warren RF, Wickiewicz TL (1987). Triceps rupture. A case report and literature review. Am J Sports Med.

[7] Searfoss R, Tripi J, Bowers W (1976). Triceps brachii rupture: case report. J Trauma.

[8] Sherman OH, Snyder SJ, Fox JM (1984). Triceps tendon avulsion in a professional body builder. A case report. Am J Sports Med.

[9] Bennett BS (1962). Triceps tendon rupture: case report and a method of repair. J Bone Joint Surg.

[10] Penhallow DP (1910). Report of a case of ruptured triceps due to direct violence. NY Med J.

[11] Duchow J, Kelm J, Kohn D (2000). Acute ulnar nerve compression syndrome in a powerlifter with triceps tendon rupture—a case report. Int J Sports Med.

[12] Sollender JL, Rayan GM, Barden GA (1998). Triceps tendon rupture in weight lifters. J Shoulder Elbow Surg.

[13] Miles JW, Grana WA, Egle D, Min KW, Chitwood J (1992). The effect of anabolic steroids on the biomechanical and histological properties of rat tendon. J Bone Joint Surg Am.

[14] van Riet RP, Morrey BF, Ho E, O’Driscoll SW (2003). Surgical treatment of distal triceps ruptures. J Bone Joint Surg Am.

[15] Müller LP, Hollinger B, Burkhart KJ (2016). Expertise Ellenbogen.

[16] Mica MC, van Riet R (2018). Triceps tendon repair. JBJS Essent Surg Tech.

[17] Viegas SF (1990). Avulsion of the triceps tendon. Orthop Rev.

[18] Lange M, Regauer M, Bocker W, Ockert B (2017). Triceps tendon rupture : double-row repair and overview of alternative techniques. Unfallchirurg.

[19] Bava ED, Barber FA, Lund ER (2012). Clinical outcome after suture anchor repair for complete traumatic rupture of the distal triceps tendon. Arthroscopy.

[20] Lempainen L, Sarimo J, Rawlins M, Heikkila J, Orava S (2011). Triceps tears in athletes: different injury patterns and surgical treatment. Arch Orthop Trauma Surg.

[21] Razaeian S, Wiese B, Zhang D, Krettek C, Meller R, Hawi N (2020). Correlation between Oxford elbow score and single assessment numeric evaluation: is one simple question enough?. J Shoulder Elbow Surg.

[22] Yeh PC, Dodds SD, Smart LR, Mazzocca AD, Sethi PM (2010). Distal triceps rupture. J Am Acad Orthop Surg.

[23] Carpenter SR, Stroh DA, Melvani R, Parks BG, Camire LM, Murthi AM (2018). Distal triceps transosseous cruciate versus suture anchor repair using equal constructs: a biomechanical comparison. J Shoulder Elbow Surg.

[24] Freislederer F, Papillo D, Glanzmann M, Scheibel M (2020). Ruptures of the distal biceps and triceps tendon. Z Orthop Unfall.

[25] Horneff JG, Aleem A, Nicholson T (2017). Functional outcomes of distal triceps tendon repair comparing transosseous bone tunnels with suture anchor constructs. J Shoulder Elbow Surg.

[26] Yeh PC, Stephens KT, Solovyova O (2010). The distal triceps tendon footprint and a biomechanical analysis of 3 repair techniques. Am J Sports Med.

[27] Mirzayan R, Acevedo DC, Sodl JF (2018). Operative management of acute triceps tendon ruptures: review of 184 cases. Am J Sports Med.

[28] Demirhan M, Ersen A (2016). Distal triceps ruptures. EFORT Open Rev.

